# Hip Manipulation Under Anesthesia for Post-Hip Arthroscopy Pericapsular Scarring: Indications and Techniques

**DOI:** 10.1016/j.eats.2023.02.036

**Published:** 2023-05-29

**Authors:** Natalie M. Gaio, Elizabeth H.G. Turner, Andrea M. Spiker

**Affiliations:** a Department of Orthopedic Surgery, University of Wisconsin—Madison, Madison, Wisconsin, U.S.A.; b Department of Orthopedic Surgery, Henry Ford Hospital, Detroit, Michigan, U.S.A.

## Abstract

Hip arthroscopy has become an increasingly common procedure with expanding indications over the last several decades. With the increase in number of procedures performed a complication profile has emerged, although there is yet to be a formal classification system for complications. The most cited complications include lateral femoral cutaneous nerve neuropraxia, other sensory deficits, chondral or labral iatrogenic damage, superficial infection and deep vein thrombosis. One complication that has not yet been well documented in the literature is pericapsular scarring/adhesions resulting in decreased hip range of motion and function. If this complication is noted to persist after adequate impingement resection and a rigorous post-operative physical therapy regimen, the senior author has addressed this with a hip manipulation under anesthesia. Therefore, this techniques paper aims to describe pericapsular scarring as a post hip-arthroscopy condition which may cause pain and demonstrate our technique to address this diagnosis through hip manipulation under anesthesia.

## Introduction

The use of hip arthroscopy has continued to grow over the past decades, in part, due to expanding indications, improved techniques and instrumentation and increased exposure of residents and fellows.^1^ Common indications for hip arthroscopy include femoroacetabular impingement syndrome (FAIS) from underlying cam and/or pincer morphology and labral pathology.^2^^,^^3^ The number of hip arthroscopic procedures has been estimated to have increased more than 600% from 2005 to 2010, with an increase of 250% from 2007 to 2011.^4^^,^^5^ From 2011 to 2018, the incidence of hip arthroscopy in patients with femoroacetabular impingement and labral pathology increased by 85%.^6^ The most recent numbers do show that the exponential growth may be plateauing at a now continued high rate.^7^ With an increasing number of hip arthroscopic procedures performed comes an increase in the associated complications. Although there is no universal grading scheme for complications related to hip arthroscopy surgery, the most commonly cited complications include neuropraxia, iatrogenic labral or chondral damage, inadequate resection resulting in continued impingement versus overresection resulting in instability, heterotopic ossification and less commonly, damage to the vasculature of the femoral head resulting in avascular necrosis.^5^ A recent large prospective multicenter study cited an overall complication rate of 8.3%, with the most common complications noted to be lateral femoral cutaneous nerve neuropraxia, other sensory deficits, chondral or labral injury, superficial portal infection, deep vein thrombosis, heterotopic ossification, or femoral neck stress fracture.^8^ Although these aforementioned complications have been previously documented in the literature, there is a paucity of information related to postoperative pericapsular adhesions resulting in stiffness of the hip after surgery, even in the setting of adequate impingement resection. Therefore, this technical note aims to describe pericapsular scarring as a post-hip arthroscopy condition, which may cause pain and demonstrate our technique to address this diagnosis through hip manipulation under anesthesia. Fortunately, in our experience, when patients have pain related to postoperative pericapsular scarring, they have achieved resolution of pain with a manipulation under anesthesia of the hip.

## Surgical Technique

### Indications and Diagnosis of Pericapsular Scarring

The hip is a complex multiaxial ball and socket joint that attributes its stability to a combination of bony anatomy, acetabular labrum, articular cartilage, ligamentous hip capsule, and surrounding musculature.^5^^,^^9^ The anatomy results in absolute limits to motion, which have been found to be on the order of 120° flexion, 10° extension, 45° abduction, 25° adduction, 15° internal rotation, and 35° external rotation.^9^ A normal gait pattern typically uses 40-50° of hip rotation, 35° hip flexion and 10° hip extension.^9^ When addressing the hip arthroscopically, there have been two major compartments described.^10^ There is the central compartment, which is composed of the lunate cartilage, acetabular fossa, ligamentum teres, and the loaded articular surface.^10^ The second compartment is the peripheral compartment, which is composed of the unloaded cartilage of the femoral head, the femoral neck with medial, anterior, and lateral synovial folds (Weitbrecht’s ligaments), and the articular capsule, which is divided into three distinct ligaments (iliofemoral ligament, ischiofemoral ligament, and femoral arcuate ligament).^10^ When work is done in the peripheral compartment to address cam type impingement, it is our belief that pericapsular scarring can occur. In the postoperative setting, this can result in either a decreased range of motion and function or no improvement in preoperative range of motion with continued hip symptoms.

In evaluating patients with residual decreased range of motion or function postoperatively, it is important to take into consideration several different etiologies. First, inadequate resection resulting in ongoing impingement is a well-documented post-hip arthroscopy condition that can account for continued anterior hip symptoms.^5^ Intraoperative evaluation, as well as radiographs, arthroscopic images, and postoperative radiographs should be scrutinized. It is the senior author’s practice to take the hip through dynamic flexion and extension at the time of the cam resection to ensure no ongoing impingement exists, confirmed with fluoroscopy and direct visualization with the arthroscope. Intraoperative arthroscopic images and fluoroscopy images can represent this dynamic assessment that took place. Further imaging can be considered in the form of an magnetic resonance image (MRI) arthrogram. An arthrogram is favored over a nonarthrogram MRI in patients who have had prior surgery. This may be helpful to rule out capsular dehiscence or labral retear, if suspected. However, if performed early after surgery (within 6 months of hip arthroscopy), images may be difficult to interpret and provide little value. Therefore, if pericapsular scarring is favored to be the cause of loss of motion, advanced imaging is not routinely obtained in the early postoperative period.

**Fig 1:**
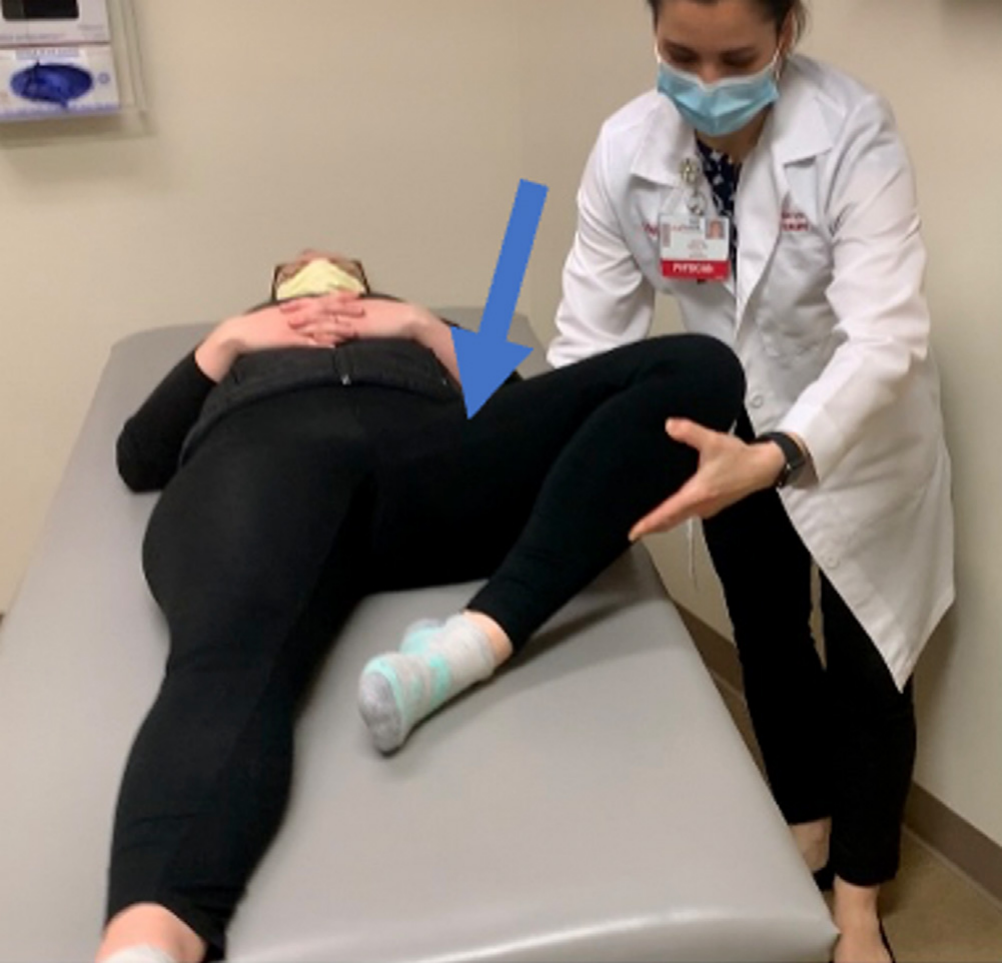
Demonstration of the butterfly position on the left side, knee flexed and hip abducted which can reproduce anterior hip pain, as indicated by the arrow, in diagnosis of pericapsular scarring postoperatively. Ideally, when evaluating the patient in clinic, both feet should be simultaneously placed in the butterfly position so that the pelvis does not rotate.

In our experience, postoperative pericapsular scarring presents as pain in the anterior hip, but patients tend to describe it as “different” from their preoperative anterior hip pain. Patients describe the pain as more superficial. The positions at which it occurs can overlap with classic impingement positions; namely, deep flexion or flexion plus adduction and internal rotation (FADIR). However, the “butterfly” position—ideally with the soles of the feet together to level the pelvis, knees flexed, and hips abducted, will also replicate the pain, and the affected side will not abduct as far to the table. With this position, patients will note a replication of the pain due to pericapsular scarring and will describe motion that is more limited than the unaffected side (Fig 1).

Our standard hip arthroscopy postoperative protocol involves the initiation of physical therapy within 1-5 days of surgery with our hip-specialized physical therapists. The published protocol for our institution can be found in Appendix A. Depending on the specific intraoperative work performed, the rehabilitation protocol can vary; however, generally weight bearing is progressed from 20% body weight with the use of crutches to full weight bearing, as gait is normalized and pain free over the course of the first 2-3 weeks postoperatively. For the first 6 weeks, there are range of motion restrictions; specifically no external rotation beyond 30° and no hip hyperextension, with the goal of protecting the labral and capsule repair during the early postoperative period. At the 6-week mark, we encourage stretching and emphasize strengthening. At 12 weeks, we reintroduce impact activity and progression of sports-specific activity. We believe it is important that all patients participate and complete a rigorous course of physical therapy before additional measures are considered to address ongoing loss of motion. However, if an adequate physical therapy regimen has been performed without improvement, hip manipulation under anesthesia is an option that we discuss with patients.

### Anesthesia for Manipulation

The post-hip arthroscopy hip manipulation procedure occurs in the operating room. Patients have an IV placed in the preoperative area for administration of IV sedation. On the continuum of depth of sedation, these cases are performed somewhere between deep sedation and general anesthesia, most often favoring general anesthesia, according to the American Society of Anesthesiologists definition.^11^ This has typically been done using a combination of midazolam, fentanyl, and propofol; although exact combinations may differ depending on the anesthesia provider and the patient. Bag mask ventilation is used as needed for respiratory support (Table 1). The procedure itself takes less than 10 minutes per hip, so giving the anesthesia team appropriate estimates of procedure length allow them to titrate their sedation medications appropriately.

**Table 1:** Equipment Required

• Bag mask ventilation
• Traction Table∗

∗ Stryker Pivot Guardian Distraction System with post or Smith and Nephew Traction table with post (in order to pull steady, controlled traction for just a couple seconds; whereas the senior author’s preference is to otherwise perform hip arthroscopy post-free).

### Patient Positioning and OR Setup

The patient is placed supine on a traction table with bony prominences well padded. While the senior author uses exclusively post-free traction for the hip arthroscopy surgery, she does use a post to pull gentle traction at the beginning of this procedure before manipulating the hip (Table 1). Antibiotics are not indicated in the absence of a planned incision and are, therefore, not administered.

### Manipulation Procedure

**Fig 2:**
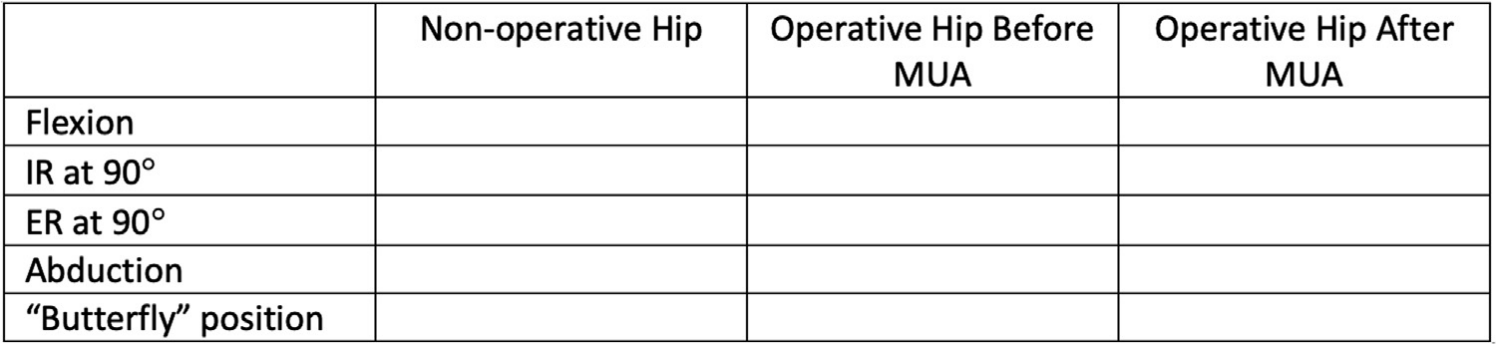
Table used to document range of motion once the patient is sedated. It is important that an assistant hold the pelvis stable during assessment of motion, as well as during the manipulation procedure, while another assistant writes the degrees of flexion down. MUA, manipulation under anesthesia.

**Fig 3:**
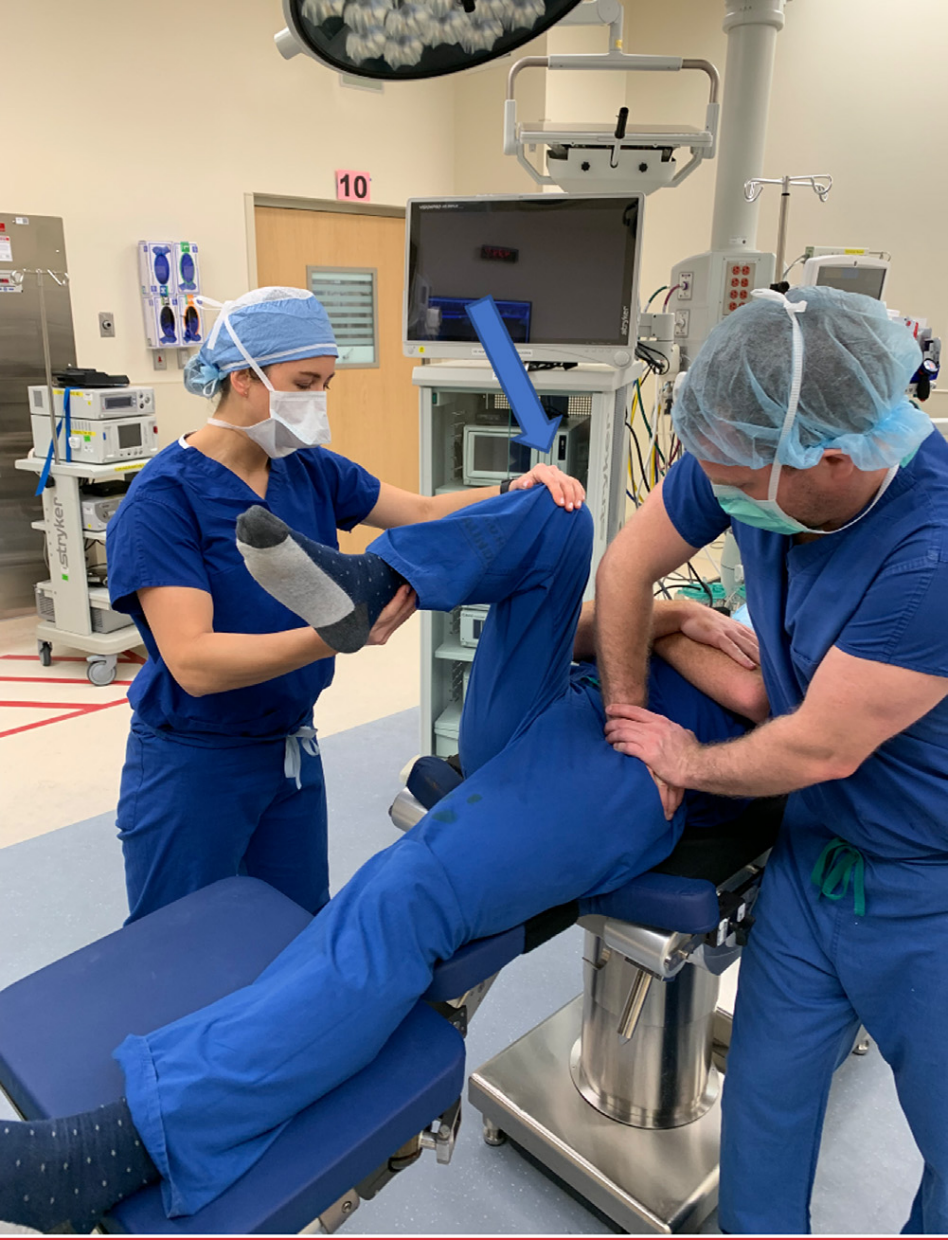
The first maneuver of hip manipulation under anesthesia is gentle hip flexion. This is performed with the patient in the supine position on the operative table. An assistant, standing on the contralateral side, should hold the pelvis stable by pushing down on the iliac crests. Flexion is performed by flexing both the hip and knee of the affected side, placing one hand on the ankle to promote flexion and one hand on the knee for control as indicated by the arrow. Flexion is performed in the neutral position (no adduction).

**Fig 4:**
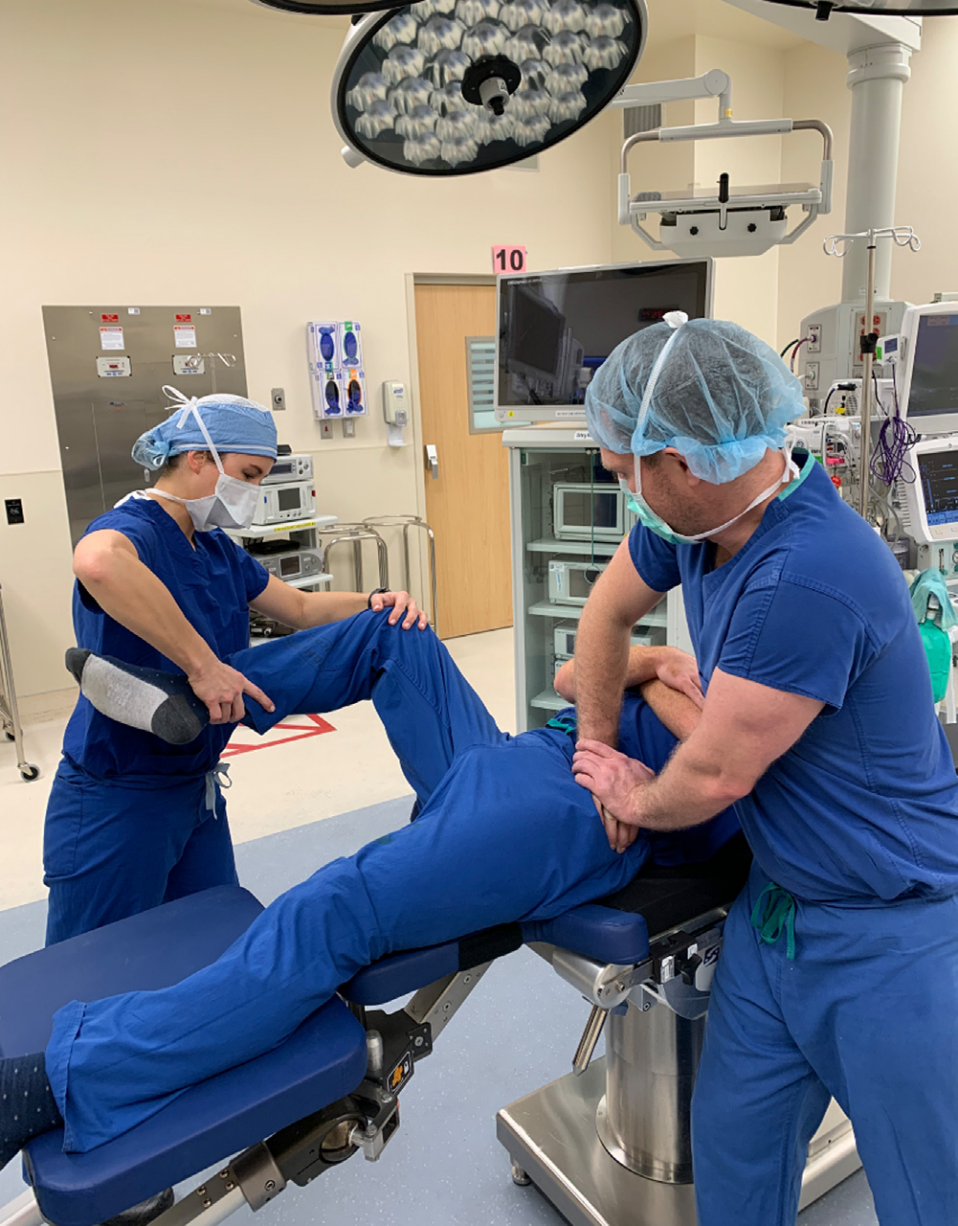
The second maneuver utilized in hip manipulation focuses on external rotation of the affected hip with a fluid motion between neutral hip flexion, while transitioning into extension. An assistant is still holding the pelvis level. The hip and knee are carefully extended in a controlled manner, supporting the knee and lower leg.

**Fig 5:**
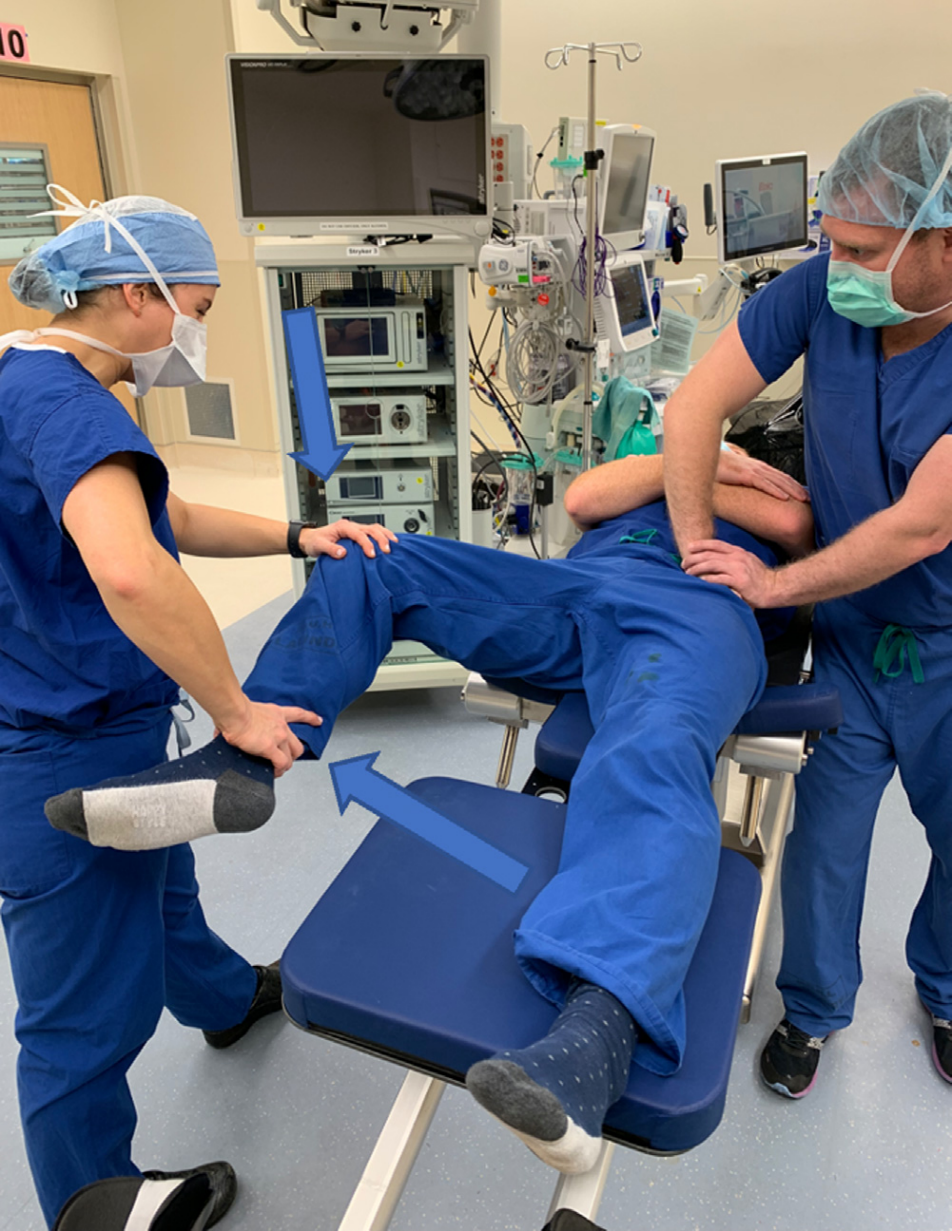
As the leg is transitioned from flexion into extension, focus is placed on abduction with external rotation of the affected hip, while the pelvis is stabilized. This is done by placing downward pressure on the knee and calf, as indicated by the arrows, while maintaining the externally rotated position described in Fig 3.

**Fig 6:**
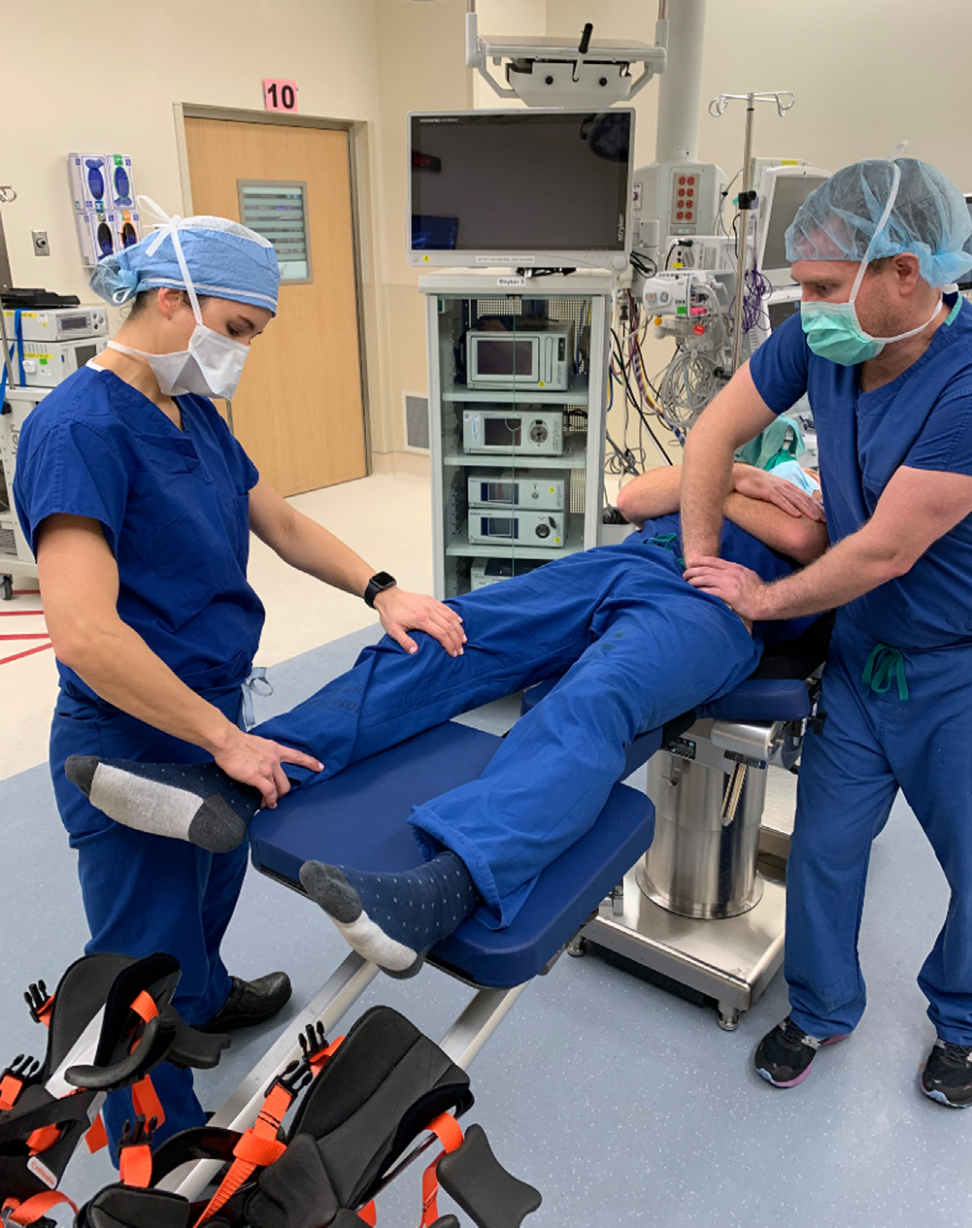
Finally, the hip is brought back into full extension. This is performed with one hand on the ankle to control extension and the contralateral hand placed under the knee for support. The full sequence of maneuvers is then repeated several times using fluid movements and applying constant gentle pressure, with an assistant helping to maintain a stable pelvis.

Premanipulation ranges of motion of both the operative and nonoperative hip are recorded (Fig 2). This includes hip flexion, hip internal rotation at 90°, hip external rotation at 90°, and hip abduction with external rotation or the “butterfly” position (Fig 1), measured as distance of the lateral thigh from the top of the bed, measured in “fists” or centimeters. The hip is then taken through a series of maneuvers (Video 1). This includes gentle flexion (Fig 2), and then the manipulation focuses mainly on externally rotating (Fig 3) and abducting (Fig 4) the hip in a fluid motion with simultaneous hip circumduction, with the pelvis stabilized by an assistant (Fig 5). Finally, the hip is brought back into full extension (Fig 6). The hip is taken through this series of maneuvers several times using gentle consistent pressure (Table 2). Postmanipulation range of motion measurements are taken and recorded as in Figure 2.

**Table 2:** Pearls and Pitfalls

Pearls • Pericapsular scarring pain is often elicited by the “butterfly” position (Fig 1), and is more superficial in comparison to preoperative impingement pain. • Have an assistant present to hold the pelvis stable during manipulation.
• Place gentle but consistent pressure on the leg during manipulation maneuvers.
• Take the hip through manipulation maneuvers several times, providing increased stretch each time. • Focus on abduction, external rotation, circumduction as most post operative pericapsular scarring occurs medially.
Pitfalls • Avoid impingement positions, as, theoretically, the labrum could retear in extreme impingement positions.
• Avoid using excessive force that could cause damage to previous labral or capsular repair or musculature strain. • While the senior author uses exclusively post-free traction for hip arthroscopy, a post is used very briefly for this procedure to gently pull the hip into traction before the manipulation procedure begins.

In patients who are limited by both pain and range of motion, we also offer hip manipulation to be performed in conjunction with fluoroscopically guided intra-articular injections. It is our goal to improve pain, so that ongoing stretching and use of the hip can be performed without deterrence by pain. Although most often a concomitant intra-articular injection is not needed, if the patient is at least 3 months out from surgery and desires this be performed simultaneously, the area is prepped in a sterile manner, and an 17 gauge spinal needle is directed into the hip capsule under fluoroscopic guidance. Intra-articular location is then confirmed by injecting up to 1 mL of iohexol solution. Once intra-articular location is confirmed, the hip is injected using a combination of 4 cc 10 mg/mL triamcinolone, 2 cc 1% lidocaine without epinephrine, and 2 cc 0.25% bupivacaine. The needle is then withdrawn, and the injection site is covered with a small bandage.

### Postoperative Protocol

This manipulation under anesthesia procedure is performed on an outpatient basis. The patient is allowed to bear full weight, as tolerated, immediately following the procedure, and there are no specific activity restrictions. Physical therapy is prescribed and encouraged postoperatively in order to maintain motion gained during the manipulation.

## Discussion

Although the use of hip manipulation under anesthesia following hip arthroscopy has not been widely documented in the literature, there are other indications for which hip manipulation under anesthesia has been performed. This dates back to the 1980s when a study was published documenting joint manipulation in head-injured patients to prevent heterotopic ossification and maintain range of motion.^12^ In this study, 11 hips were included. Four had very little motion prior to manipulation, and minimal improvement was seen. However, of the remaining 7 there was an average of 52° increase in range of motion, and six patients had greater than 85° gained.^12^ It was documented that the manipulation involved hip flexion with internal and external rotation and extension over the edge of the table.^12^ A second study published more recently in 2016 was a systematic review, in which treatment options for adhesive capsulitis of the hip were reviewed, including manipulation under anesthesia.^13^ In this study, hip manipulation was noted to result in general improvements in pain and range of motion. Therefore, the technique of hip manipulation under anesthesia in itself is not novel and has been documented to result in improvements for pathology outside of post-hip arthroscopy pericapsular adhesions. However, when used in the context of post-hip arthroscopy, we suggest differences in the previously documented manipulation techniques. These differences include avoiding impingement motions or positions and focusing on stretching of the areas beneath the rectus femoris and hip flexors, where scarring may have occurred in the postoperative time period. This is especially targeted through the “butterfly” position and dynamic hip external rotation with simultaneous abduction, circumduction, and extension. This intervention is particularly advantageous for targeting scar tissue, as it does not require an additional incision, which would be counterproductive to scar formation. This procedure is also associated with minimal risk given the short duration and minimal sedation necessary (Table 3). However, it does still rely on patient participation in therapy and is best for pericapsular, as opposed to intracapsular scarring (Table 3). While hip arthroscopy has been previously documented to result in increased hip range of motion (15°-20° on average), it has been noted that some patients still struggle to regain this motion postoperatively despite adequate surgical technique and therapy.^14^ We propose that pericapsular adhesions and scarring should be included in the differential for patients who have residual lack of range of motion after hip arthroscopy, and that when diagnosed, hip manipulation under anesthesia can be a viable treatment option.

**Table 3:** Advantages, Risks, and Limitations

Advantages • Short duration of procedure
• No incision required, which could be counterproductive in forming additional scar
Risks • Involves the use of sedation and its established risks Limitations • Relies on patient participation in therapy • Not as effective on intracapsular scarring compared to pericapsular scarring

## Figures and Tables

**Appendix Table 1A:** Hip Arthroscopy Postoperative Rehabilitation Protocol

	Weeks Post-op	Goals	Precautions	Criteria Necessary to Advance to Next Phase
Phase 1	1-3	• Protect surgical hip with limited weight bearing • Restore normal hip range of motion (ROM) within ROM restrictions • Normalize gait • Restore leg control	• Avoid for six weeks: • External rotation past 30° • Hip hyperextension • Abduction past 45° • May initially bear 20% body weight, flat foot weight bearing, for all procedures	• Normal gait on level indoor surfaces without pain nor assistive device • Functional ROM without pain • Good leg control at low velocity movement
Phase 2	3-6	• Wean off crutches • Regain/Improve muscular control • Single leg stance control • Good control and no pain with functional movements • More aggressive stretching begins at 6 weeks post-op	• Postactivity soreness should resolve within 24 hours • No ballistic or forced stretching • Caution with repetitive hip flexion activities, such as treadmill or Stairmaster	• Normal gait on all surfaces • Functional movements without unloading affected leg • 10 single leg or split squats, balance testing
Phase 3	6-20	• Improve muscular strength and endurance • Good control, no pain with work or sport specific activities, including impact • Impact activity begins at 12 weeks post-op	• Postactivity soreness should resolve within 24 hours • Caution with forceful hip flexion activities, such as kicking or sprinting	• Normal gait on all surfaces • Dynamic neuromuscular control • Pass progressive testing

## Supplementary Data

ICMJE author disclosure forms

Video 1 A demonstration of the hip manipulation under anesthesia technique. Both the nonoperative and operative hips are first examined, and range of motion is documented as demonstrated in Fig 2. The operative hip is then briefly pulled into traction, then taken through range of motion, including hip flexion, followed by a combination of abduction, external rotation, and hip extension with simultaneous hip circumduction. An assistant stabilizes the pelvis throughout these movements. This sequence is performed several times, applying gentle pressure with each maneuver. Postmanipulation range of motion measurements are then recorded.
